# Reluctant knee: understanding and managing stiffness after total knee arthroplasty

**DOI:** 10.1007/s00264-026-06905-0

**Published:** 2026-06-25

**Authors:** Sakeena Hosanee, Nnena Elebo, Nkhodiseni Sikhauli, Jurek Rafal Tomasz Pietrzak

**Affiliations:** 1 Division of Orthopaedics, University of the Witwatersrand, Johannesburg, South Africa; 2 Arthroplasty Unit, Charlotte Maxeke Johannesburg Academic Hospital, Johannesburg, South Africa

**Keywords:** Total knee arthroplasty, Postoperative knee stiffness, Arthrofibrosis, Range of motion limitation, Manipulation under anaesthesia

## Abstract

**Purpose:**

Post-total knee arthroplasty (TKA) stiffness remains a clinically important cause of pain, restricted range of motion (ROM), functional limitation, and reintervention. This narrative review synthesises current evidence on the pathophysiology, evaluation, and staged management of post-TKA stiffness, with particular emphasis on manipulation under anaesthesia (MUA).

**Methods:**

A narrative review was performed using literature relating to post-TKA stiffness, arthrofibrosis, diagnostic evaluation, rehabilitation, MUA, lysis of adhesions, and revision TKA. Evidence was synthesised thematically to support a practical diagnostic and management framework.

**Results:**

Acquired idiopathic stiffness occurs in approximately 3.6% to 4.0% of TKAs, while MUA is required in roughly 2.6% to 3.6% of cases. The available literature supports a diagnosis-of-exclusion approach, in which infection, component malposition, instability, and other secondary causes are excluded before arthrofibrosis is diagnosed. MUA remains the principal first-line procedural intervention once early conservative measures fail. Earlier MUA, generally within 6–12 weeks, is associated with greater ROM gain than delayed intervention, although outcomes remain influenced by baseline ROM severity, patient selection, and the quality of post-manipulation rehabilitation.

**Conclusion:**

Post-TKA stiffness is a heterogeneous complication requiring structured evaluation and staged management. When MUA fails, escalation to arthrolysis or revision TKA should be individualised and guided by the distinction between persistent soft-tissue fibrosis and correctable mechanical pathology. A practical algorithm is proposed to support diagnosis, timing of intervention, and escalation after failed MUA.

**Supplementary Information:**

The online version contains supplementary material available at https://doi.org/10.1007/s00264-026-06905-0.

## Introduction

Total knee arthroplasty (TKA) is one of the most successful procedures in orthopaedic surgery, with a patient satisfaction rate of around 80% [1]. Using the KOOS (Knee injury and Osteoarthritis Outcome Score) based outcomes, baseline-to-1-year improvements of approximately 36.8 points for pain, 30.3 points for function, and 40.7 points for quality of life have been reported after TKA [2]. A meaningful minority of 10–20% of patients still remain dissatisfied [3, 4]. Contributors to this rate include persistent pain in 41% of dissatisfied patients, with 26% reporting functional limitation including knee stiffness [5]. In a meta-analysis by Tibbo et al., which included 35 studies and 48,873 patients, the mean prevalence of acquired idiopathic stiffness was approximately 4% [6]. In a large institutional cohort, Owen et al. reported acquired idiopathic stiffness in 3.6% of TKAs [7].

Stiffness after TKA is disabling, as it compromises activities that depend on functional knee flexion and extension, such as gait, stair negotiation, sitting and rising from a chair, and kneeling, which may ultimately necessitate further procedures such as manipulation under anaesthesia (MUA), arthrolysis, or revision TKA. For this reason, post-TKA stiffness remains an important source of morbidity, readmission, reintervention and resource use [1, 8].

## Pathophysiology and aetiology of post-TKA stiffness

Post-TKA stiffness is multifactorial and reflects the interaction of pre-operative patient characteristics, intra-operative technical factors, and post-operative biological and rehabilitative responses [1].

### Patient-specific risk factors

A study by Lee et al. which included 122 knees found that, among patients with preoperative knee stiffness undergoing TKA, better preoperative range of motion (ROM) was significantly associated with better postoperative ROM [9]. A large meta-analysis found prior knee surgery approximately doubled the odds of requiring MUA after primary TKA [10]. Contemporary epidemiological data also support younger age as a reproducible risk factor. In pooled analysis, Patel et al. found Black race to be a significant risk factor for MUA, whereas female sex was not significant [10]. By contrast, large database evidence from Rullán et al. suggested higher odds of MUA in younger patients, women, and Black or Hispanic patients after primary TKA [11]. The role of BMI is less consistent, Tibbo et al. reported a higher pooled prevalence of acquired idiopathic stiffness in patients with BMI ≥ 30 kg/m^2^ than in those with BMI < 30 kg/m^2^ (5% vs 2%; p = 0.027), whereas Owen et al. found BMI ≥ 30 kg/m^2^ to be associated with a lower adjusted hazard for acquired idiopathic stiffness in a single-institution multivariable model [6, 7]. Additionally, Owen et al. identified diabetes as an independent risk factor for acquired idiopathic stiffness and subsequent MUA, with an adjusted hazard ratio of 1.49 (95% CI 1.11–1.99) [7]. These data suggest that pre-operative risk is best understood as cumulative rather than attributable to any single variable alone [1] as described in Table 1.

**Table 1:** Risk factors for post-TKA stiffness and mua requirement

Category	Risk Factor	Supporting Evidence	Clinical Implication
Patient-Specific Factors	Restricted pre-operative ROM	Consistent across multiple studies (Wiederhold 2026; Patel 2025)	Strongest modifiable pre-operative predictor; optimise flexion before surgery where possible
	Prior ipsilateral knee surgery	OR ~ 2 × for MUA (Patel et al., 2025)	Thorough pre-operative counselling; heightened post-operative surveillance
	Younger age (< 65 years)	Significant in pooled analysis (Patel 2025; Rullán 2022)	Higher functional demands may increase clinical detection threshold
	Black race	Significant risk factor in pooled meta-analysis (Patel 2025)	Higher-risk subgroup in current evidence base
	Hispanic ethnicity	Supported by Rullán 2022 database study, not Patel pooled analysis	Higher-risk subgroup in large administrative data; requires further confirmatory evidence
	Type 2 diabetes mellitus	Significant for AIS/MUA in Owen 2021; not significant in pooled MUA analysis by Patel 2025	May contribute to fibrosis risk, but should not be treated as a uniform pooled predictor of MUA
	Smoking	Significant in meta-analysis (Patel 2025)	Cessation counselling pre-operatively
	BMI ≥ 30 kg/m^2^	Conflicting: higher AIS (Tibbo 2019) but lower HR in single-centre model (Owen 2021)	Inconsistent; do not use in isolation for risk stratification
	Female sex	Significant in Rullán 2022 database study; NS in Patel 2025 meta-analysis	Discordant evidence; should not be considered a pooled established predictor
	Prior DVT	Higher prevalence in MUA cohort (6.8% vs 3.8%, DeFrance 2022)	Thromboembolic history may influence peri-procedural anticoagulation planning
Operative and Technical Factors	Component malrotation	Well-established cause of mechanical stiffness (Wiederhold 2026; Singh 2019)	Systematic exclusion mandatory before diagnosing arthrofibrosis; CT if suspected
	Anterior overstuffing/patellofemoral maltracking	Recognised cause of restricted flexion (Wiederhold 2026)	Careful patellar tracking assessment at index procedure
	Flexion–extension gap imbalance	Directly restricts arc of motion post-operatively (Wiederhold 2026)	Meticulous ligament balancing; intra-operative gap assessment
	Inappropriate tibial slope	Associated with restricted extension/posterior impingement (Singh 2019)	Restore native tibial slope; avoid posterior slope deficiency
	Conventional vs robotic/computer-assisted TKA	Lower MUA odds with technology-assisted TKA (Rullán 2022)	Precision alignment may reduce risk; requires further prospective evidence
Post-operative/Biological Factors	Pre-MUA flexion contracture ≥ 5°	Associated with repeat MUA and revision (Debbi 2025)	Key prognostic marker once MUA is being considered
	Lower pre-MUA flexion (severity of contracture)	Lower pre-MUA ROM → lower probability of durable success (Debbi 2025; Akhtar 2024)	Severity at time of MUA inversely predicts outcome
	Delayed rehabilitation or inadequate physiotherapy	Adhesion maturation; progressive ROM loss (Chalmers 2025; Ramos 2023)	Structured early physiotherapy is critical; avoid both aggressive and inadequate regimens
	Occult PJI masquerading as arthrofibrosis	Recognised mimic; must be excluded before MUA (Ramos 2023; Wiederhold 2026)	Systematic pre-MUA infection screen (ESR, CRP, aspiration if indicated)
	TGF-β/myofibroblast-driven fibrosis	Central molecular mechanism (Ramos 2023; Morrey 2017)	Temporal biology supports early intervention before collagen cross-linking matures

Risk factors classified by domain: patient-specific, operative/technical, and post-operative/biological. Evidence quality varies from pooled meta-analytic to single-centre observational..Abbreviations: ROM = range of motion; AIS = acquired idiopathic stiffness; MUA = manipulation under anaesthesia; HR = hazard ratio; OR = odds ratio; NS = not significant; PJI = periprosthetic joint infection; TGF-β = transforming growth factor beta; CT = computed tomography

### Operative technical risk factors and infection

Technical causes of stiffness include component malrotation, coronal malalignment, patellofemoral maltracking, anterior overstuffing, inappropriate tibial slope, and flexion–extension gap imbalance, all of which can impair knee kinematics and restrict ROM [4, 12]. Clinically, subacute or culture-negative periprosthetic joint infection also remains an important mimic and should be excluded systematically before labelling a patient as having arthrofibrosis [1, 12].

### Molecular and biological mechanisms

At a molecular level, arthrofibrosis appears to represent an exaggerated wound-healing response characterised by persistent myofibroblast activation, excess type I collagen deposition, and reduced matrix remodelling [1, 12]. TGF-β is the best-established central pathway and drives fibroblast-to-myofibroblast differentiation, collagen synthesis, and persistence of a pro-fibrotic extracellular matrix [1, 12]. Additional mediators implicated in fibrosis progression include IL-6, IL-17, TNF-α, Wnt/β-catenin, LOX, PAI-1, and NF-κB-related signalling [1, 12]. In a rabbit contracture model, Morrey et al. demonstrated that inflammatory genes predominated during the earliest post-injury period, whereas extracellular matrix and remodelling pathways became more prominent later, suggesting a temporally staged biological process rather than a single static fibrotic event [13]. This mechanistic framework helps explain why developing stiffness is generally more responsive to early intervention than established late fibrosis, once collagen cross-linking and scar maturation have progressed [1, 13].

## Clinical evaluation of post-TKA stiffness

### Definition and classification

Post-TKA stiffness remains inconsistently defined in the literature, and this lack of uniformity has contributed to wide variation in reported incidence, treatment thresholds, and outcome reporting [1, 6]. An international consensus by Kalson et al. has defined fibrosis of the knee as restricted ROM in flexion and/or extension due to soft-tissue fibrosis that was not present preoperatively, after excluding other causes of stiffness such as component malposition, incorrect sizing, hardware, ligament deficiency, infection, pain, CRPS, or other specific causes. The same group graded severity as mild, moderate, or severe according to the degree of flexion loss and extension deficit (see Table 2) [14].

**Table 2:** Classification of severity of knee fibrosis post TKA [14]

Severity	Flexion	Extension deficit
Mild	90°–100°	5°–10°
Moderate	70°–89°	11°–20°
Severe	< 70°	> 20°

### Clinical evaluation

Clinical evaluation begins with a structured history and examination focused on the time course, progression of stiffness, pain pattern, and rehabilitation trajectory, to distinguish early postoperative fibrosis from infection, implant-related problems, or pain-mediated limitation. Examination should document active and passive range of motion, the pattern of flexion and/or extension loss, end feel, patellar mobility, extensor mechanism function, joint stability, and signs of swelling, warmth, or effusion. Differentiating active from passive limitation is particularly important, because extensor mechanism dysfunction, pain inhibition, or patellofemoral problems may produce apparent stiffness without representing true capsular fibrosis [1, 12].

### Diagnostic work-up

The diagnostic work-up of the stiff TKA should follow a structured sequence aimed at excluding reversible pathology before arthrofibrosis is diagnosed. Serological investigations should then be performed to exclude infection. CRP and ESR remain first-line screening tests for PJI [15]. If inflammatory markers are elevated, or if suspicion persists despite equivocal serum testing, joint aspiration with synovial leukocyte count, differential, and culture is indicated as per the MSIS/ICM diagnostic criteria [16].

Imaging begins with plain radiographs, to assess component position, alignment, patellar height, joint line, loosening, gross overstuffing, and heterotopic ossification. When radiographs are inconclusive and a mechanical cause remains suspected, CT with 3D reconstruction is particularly valuable [12]. SPECT/CT has also been shown to provide additional diagnostic value in the assessment of painful knee prostheses. A 2023 systematic review and diagnostic test accuracy meta-analysis of 8 studies including 308 patients reported pooled sensitivity of 0.86, and specificity of 0.90 for SPECT/CT in identifying the source of pain in painful knee prostheses [17]. It is therefore a reasonable second-line investigation when occult loosening, malalignment, patellofemoral overload, or instability is suspected in a noninfected but diagnostically unclear TKA [17, 18].

### Pain management principles

Pain assessment is essential because persistent pain after TKA may reflect biological, surgical, neuropathic, or psychosocial mechanisms rather than true capsular fibrosis. In patients with disproportionate pain, neuropathic features, or suspected CRPS, targeted pain management should precede invasive stiffness procedures [19, 20].

## Non-operative management and prevention

### Prevention and early rehabilitation principles

Prevention of post-TKA stiffness begins before irreversible fibrosis is established and depends on minimising inflammation while preserving early motion [1, 21]. Once stiffness starts developing, the goals are pain control, swelling reduction, restoration of functional motion and maintenance of mobility required for activities of daily living (ADL) without provoking further inflammatory insult [12]. Multimodal, opioid-sparing analgesia, cryotherapy, elevation, compression and pain-adapted physiotherapy with frequent short-duration ROM stimuli are therefore preferred over forceful stretching or highly aggressive passive mobilisation [12, 21].

### The role of CPM

Routine CPM (continuous passive motion) after TKA has not demonstrated clinically meaningful benefit in ROM, pain, swelling, or functional recovery when compared with early mobilisation protocols. Boese et al., in a randomised trial of 160 TKA patients, found no clinically significant benefit of CPM compared with no CPM in early ROM [22]. Another study looked specifically at patients who underwent MUA for post-TKA stiffness finding that adjunctive inpatient CPM did not improve the maintenance of ROM gains compared with MUA alone[23].

### Pharmacological prevention

Pharmacological prevention remains promising but is not yet standardised. Observational studies suggest possible benefit from celecoxib- and dexamethasone-based strategies, but regimen, dose and duration remain uncertain [1, 21, 24]. Non-operative treatment should therefore be viewed as a biologically and functionally important early phase of care, but one that requires close reassessment so that ineffective nonoperative management does not delay timely escalation to MUA when motion fails to recover.

## Manipulation under anaesthesia (MUA)

MUA remains central to the management discussion because it is the most widely used first-line procedural treatment once early conservative measures have failed, yet important questions persist regarding optimal timing, patient selection, durability of benefit, adjunctive treatments, and when escalation to revision becomes justified [21, 25].

### Patient selection

Appropriate patient selection is essential, because MUA is most effective when stiffness is primarily soft-tissue mediated and when mechanical, infective or major pain-driven causes have been excluded [1, 12]. In practical terms, candidates are typically patients with persistent limitation of flexion despite supervised rehabilitation, usually in the setting of a relatively “soft” endpoint and without radiographic evidence of malposition, instability, loosening, or heterotopic ossification [12, 21].

### Indications and range of motion thresholds

Although no universal ROM threshold exists, most studies use postoperative flexion failure somewhere between < 90° and < 95° in the first six to 12 weeks as a practical trigger for considering MUA [12, 25]. Some centres use more aggressive thresholds. Sirignano et al. performed MUA for failure to achieve 105° of active flexion at  six weeks, and reported equivalent longer-term PROMs and satisfaction compared with matched uncomplicated TKA controls, suggesting that earlier intervention at a higher motion threshold may be reasonable in younger or higher-demand patients [26]. Earlier postoperative ROM trajectory is also highly informative. In a cohort of 1,546 primary TKAs, LaHaise et al. found that patients discharged with flexion < 65° had an odds ratio of 17.57 for later MUA compared with those discharged at > 90°, while discharge flexion of 65°−75° and 80°−90° also remained strongly associated with subsequent manipulation [27]. These findings suggest that patient selection for MUA should not rely on a single late ROM cutoff, but rather on the trajectory of recovery, early motion milestones, and failure to progress despite appropriate therapy [26, 27].

### Risk stratification and prognostic markers

Once MUA is being considered, prognostication should shift from demographic risk enrichment to the severity and pattern of motion loss. Although younger age, prior knee surgery, smoking, and race have been associated with increased odds of requiring MUA after primary TKA, these factors should not be used as independent indications for manipulation. Instead, they may identify patients who require closer postoperative ROM surveillance.

Once a patient presents for MUA, the most clinically relevant prognostic marker may be the severity of pre-MUA motion loss itself. In a large single-institution cohort, Debbi et al. found that patients with a pre-MUA flexion contracture ≥ 5° were more likely to undergo repeat MUA and revision for stiffness, and that lower pre-MUA flexion was associated with greater risk of treatment failure [28]. DeFrance et al. further showed that patients undergoing MUA had a higher subsequent non-infectious revision rate than matched controls (7.3% vs 4.9%; p = 0.034), with persistent stiffness accounting for 64% of post-MUA revisions, reinforcing that manipulation should be offered thoughtfully rather than reflexively [3].

### Contraindications

Contraindications to MUA are equally important for patient selection. Mechanical obstruction, component malposition, extensor mechanism compromise, fracture risk and unresolved or suspected infection should obviously preclude routine manipulation until the underlying pathology has been clarified or corrected [4, 12]. Moreover, patients with predominantly pain-driven stiffness, disproportionate hypersensitivity, or poorly controlled complex pain syndromes may not respond predictably to manipulation and may deteriorate if the dominant pathology is not fibrotic restriction [21].

## Manipulation under anaesthesia: timing

Timing is one of the most important determinants of MUA efficacy after TKA, and most contemporary literature supports intervention during the early postoperative period before mature scar consolidation has occurred [21, 25]. In practice, most surgeons consider MUA between 6 and 12 weeks after index arthroplasty if flexion remains unsatisfactory despite appropriate rehabilitation, and international survey data suggest this interval has become the de facto standard of care [12, 29].

The strongest contemporary comparative evidence comes from the meta-analysis by Akhtar et al., which included 14 studies and 13,445 knees. Across included studies, most defined early MUA as occurring within three months of the index TKA. Pre-MUA flexion was higher in delayed cases, but post-MUA flexion was similar between groups. However, mean flexion gain was 32.0 degrees in the early group compared with 19.2 degrees in the delayed group, and delayed MUA was associated with significantly higher risks of surgical or medical complications and revision TKA [25]. Even so, the timing literature is not entirely one-sided. Smaller retrospective series have shown that later MUA can still produce clinically meaningful gains [30, 31].

## Manipulation under anaesthesia: technique

### Anaesthesia and patient setup

Manipulation under anaesthesia is intended to disrupt immature intra-articular adhesions and periarticular scar bands while avoiding iatrogenic injury to bone, extensor mechanism, or prosthetic constructs [12, 21]. Although technique varies somewhat between surgeons and centres, the essential principle is controlled progressive flexion of the knee under adequate anaesthesia to achieve muscle relaxation and reduce patient guarding [12, 32]. In current practice, MUA is usually performed under general or spinal anaesthesia, after which the hip is flexed and the tibia is held proximally so that force is applied with a short lever arm, thereby minimising torque across the femur and tibia in order to prevent complications [12, 33].

### The manipulation technique

The manoeuvre itself should be deliberate rather than abrupt. Steady progressive flexion is applied until palpable and sometimes audible release of adhesions is encountered, after which the knee may be cycled through flexion and extension to consolidate the gained motion [12, 33]. The process typically requires several minutes of steady, continuous loading to achieve a durable increase in motion through plastic deformation. Some authors also emphasise mobilisation of the patellofemoral articulation at the end of the procedure, particularly when anterior interval or suprapatellar fibrosis is suspected [33]. However, immediate post-MUA ROM should not be mistaken for final outcome, because some of this gain is typically lost during the early postoperative period before a more stable final range is established [32, 34].

### Peri-procedural management and rehabilitation

Technique must also be considered together with peri-procedural management. Because the gains achieved at manipulation are vulnerable to early recurrence, most protocols begin frequent supervised ROM exercises within the first 24 to 48 h, followed by a structured home exercise programme and formal physiotherapy [12, 29]. Abdel et al. also demonstrate that adjunctive peri-procedural anti-inflammatory therapy can be incorporated into a standardised technique pathway, although in their trial intravenous dexamethasone plus a 14-day celecoxib course did not improve ROM or PROMs over standard care alone [32]. Accordingly, the technical success of MUA should be viewed as a combination of three linked steps: careful low-trauma manipulation, immediate confirmation of improved motion and structured rehabilitation to preserve that gain.

### Safety considerations and technical pitfalls

Complications related to MUA are uncommon, but the technique should always be performed with awareness of fracture risk and soft-tissue vulnerability. Across modern literature, the overall complication rate is generally reported at around 1% to 2%, with most serious adverse events being rare but potentially catastrophic [21, 33].

## When manipulation under anaesthesia fails

### Reassessment after MUA failure

Failure of MUA does not automatically mandate revision TKA, but it should prompt systematic reassessment of the cause of persistent stiffness and the likely mechanism of treatment failure. In some patients, MUA fails because fibrosis has become too mature or severe for durable nonoperative disruption, whereas in others the more important problem is unrecognised mechanical pathology [1, 12].

### Arthroscopic and open lysis of adhesions

Arthroscopic or open lysis of adhesions occupies the intermediate step between failed MUA and revision TKA. Arthroscopic lysis is best suited to predominantly anterior, suprapatellar, or patellofemoral adhesions, whereas open arthrolysis is more appropriate when there is dense posterior capsular contracture, severe extension loss, or extensive scarring involving the extensor mechanism [12, 35]. In the systematic review by Haffar et al., arthroscopic lysis of adhesions improved ROM by a mean gain of 40°, although 25% of patients still required further treatment [35]. By comparison, revision TKA in the same review improved ROM from 54.6° to 82.9° for a mean gain of 28°, but with a higher burden of further intervention and generally less favourable functional outcomes than MUA or arthroscopic lysis [35]. These data support a stepwise approach in which arthrolysis may be considered before revision when stiffness is still believed to be primarily scar-mediated and no correctable implant-related cause has yet been identified.

### Revision TKA for arthrofibrosis

Revision TKA is generally reserved for cases in which a clear mechanical contributor is present or when all lesser interventions have failed to restore acceptable motion [1, 12]. A contemporary cohort by Rockov et al. analysed 42 revision TKAs performed specifically for arthrofibrosis and found significant ROM improvement at a mean follow-up of 4.5 years. Terminal flexion improved by 18.4°, extension by 6.8°, and total arc of motion by 25.2°. However, the gains were not synonymous with full functional recovery: PROMIS physical function and pain interference scores remained consistent with moderate dysfunction, reminding us that motion can improve without complete normalisation of subjective outcome. In addition, 21.4% of patients required MUA after revision, 2.4% required lysis of adhesions, and 16.7% underwent a further revision procedure, illustrating that revision for stiffness is not benign [8].

## Management algorithm

**Fig. 1:**
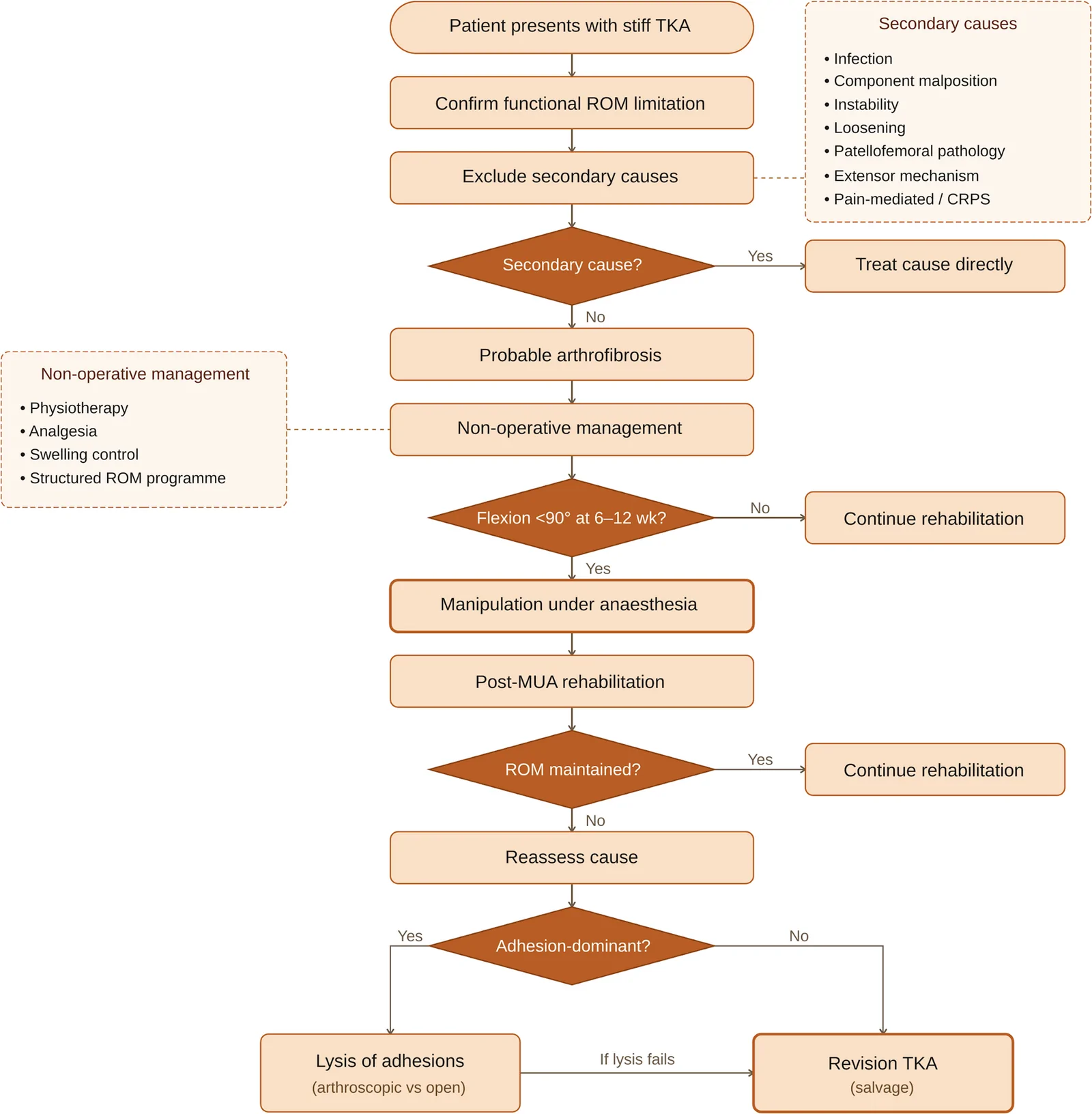
Algorithm for the assessment and management of stiffness after total knee arthroplasty (TKA). Functional range-of-motion limitation is first confirmed, and secondary causes such as infection, malposition, instability, or loosening are excluded. In the absence of secondary pathology, probable arthrofibrosis is treated with non-operative measures including physiotherapy, analgesia, swelling control, and structured rehabilitation. Patients with persistent flexion < 90° at 6–12 weeks may undergo manipulation under anaesthesia (MUA) followed by rehabilitation. Persistent or recurrent stiffness requires reassessment, with lysis of adhesions or revision TKA considered for refractory cases

When rehabilitation fails to restore an acceptable ROM trajectory, escalation should be individualised, progressing from appropriately timed MUA to arthrolysis or revision TKA according to the underlying pathology. The proposed management algorithm is summarised in Fig. 1.

## Gaps in the literature and future directions

Despite increasing interest in post-TKA stiffness, the literature remains limited by a persistent lack of uniformity in terminology, diagnostic criteria, and outcome definition [1, 6]. “Stiffness,” “fibrosis,” “acquired idiopathic stiffness,” and “arthrofibrosis” are still used inconsistently, and studies continue to apply different flexion and extension thresholds when defining the condition or indicating MUA [6, 29]. This makes direct comparison between studies difficult and likely contributes to the wide reported variation in incidence, treatment rates, and apparent success of intervention [6, 10]. Even recent meta-analyses remain dependent on heterogeneous retrospective cohorts rather than standardised prospective datasets, which limits the certainty with which pooled estimates can be translated into clinical algorithms [10, 25].

A second major limitation is that most outcome studies continue to prioritise ROM over patient-centred recovery [28, 33]. Although recent work has improved this, PROMs remain inconsistently reported, follow-up intervals are variable, and many studies do not distinguish between immediate post-manipulation gain and durable maintained motion at medium- or long-term follow-up [28, 34]. Similarly, revision-free survivorship, repeat MUA rates, cost burden, return to function, and quality-of-life outcomes are still less consistently measured than flexion alone [33, 35]. This matters because successful treatment of stiffness should not be defined merely by a goniometric threshold, but by restoration of useful function, pain reduction, and acceptable patient satisfaction [8, 26]. Abdel et al. are notable in this regard because their multicentre randomised trial incorporated KSS, KOOS, PROMIS, and SF-12, illustrating the kind of multidimensional outcome reporting that future stiffness studies should adopt more routinely [32].

Important biological and therapeutic questions also remain unresolved. Although TGF-β signalling, myofibroblast persistence, and inflammatory-fibrotic crosstalk are increasingly well described, there are still no validated clinical biomarkers that identify patients at high risk of arthrofibrosis or reliably distinguish biologically active fibrosis from other causes of stiffness [1, 12].

Pharmacologic prevention remains biologically plausible but clinically unresolved. Current evidence is insufficient to define a standard prophylactic or adjunctive regimen, and recent randomised data have shown that peri-procedural dexamethasone plus celecoxib did not improve ROM or PROMs after MUA [1, 21, 24]. Likewise, genetic predisposition, prior contralateral susceptibility, and molecular fibrosis pathways remain conceptually important but clinically underdeveloped. Future research should therefore move beyond retrospective association studies toward prospective phenotype-based cohorts and randomised trials of biologically informed prevention or adjunctive treatment.

## Conclusion

Post-TKA stiffness is a heterogeneous complication rather than a single pathological entity, and its evaluation must begin with exclusion of infection, mechanical error, and other secondary causes before arthrofibrosis is diagnosed [1, 12]. Early recognition and timely escalation to appropriately selected MUA remain central to management. When MUA fails, further escalation should be individualised: arthrolysis may be appropriate in selected adhesion-dominant cases, whereas revision TKA should generally be reserved for clearly defined mechanical failure or severe refractory fibrosis after lesser interventions have been exhausted.

## Supplementary Information

Below is the link to the electronic supplementary material.

ESM 1 (DOCX 19.7 KB)

## Data Availability

No datasets were generated or analysed during the current study.

## Abbreviations

*ADLs*: Activities of daily living
*BMI*: Body mass index
*CPM*: Continuous passive motion
*CRP*: C-reactive protein
*CRPS*: Complex regional pain syndrome
*CT*: Computed tomography
*EMBASE*: Excerpta Medica Database
*ESR*: Erythrocyte sedimentation rate
*ICM*: International Consensus Meeting
*IL*: Interleukin
*KOOS*: Knee injury and Osteoarthritis Outcome Score
*KSS*: Knee Society Score
*LOA*: Lysis of adhesions
*LOX*: Lysyl oxidase
*MEDLINE*: Medical Literature Analysis and Retrieval System Online
*MeSH*: Medical Subject Headings
*MSIS*: Musculoskeletal Infection Society
*MUA*: Manipulation under anaesthesia
*NF-κB*: Nuclear factor kappa B
*PAI-1*: Plasminogen activator inhibitor-1
*PJI*: Periprosthetic joint infection
*PRISMA-ScR*: Preferred Reporting Items for Systematic Reviews and Meta-Analyses extension for Scoping Reviews
*PROM*: Patient-reported outcome measure
*PROMIS*: Patient-Reported Outcomes Measurement Information System
*PROMs*: Patient-reported outcome measures
*ROM*: Range of motion
*SANRA*: Scale for the Assessment of Narrative Review Articles
*SF-12*: 12-Item Short Form Survey
*SPECT/CT*: Single-photon emission computed tomography/computed tomography
*TGF-β*: Transforming growth factor beta
*TKA*: Total knee arthroplasty
*TNF-α*: Tumour necrosis factor alpha
