# Supplementary material for: Reluctant knee: understanding and managing stiffness after total knee arthroplasty
Source: Int Orthop. 2026 Jun 25;50(9):2337–46. doi: 10.1007/s00264-026-06905-0 (PMC13562152; doi:10.1007/s00264-026-06905-0)
Supplement: Supplementary file 1 [file 264_2026_6905_MOESM1_ESM.docx]

# Supplementary Tables

**Supplementary Table 1: Key Studies on Incidence and Epidemiology of Post-TKA Stiffness and MUA**

| Study (Author, Year, Journal) | Study Design | Sample Size | AIS Prevalence | MUA Rate | Key Findings / Risk Factors |
| --- | --- | --- | --- | --- | --- |
| Tibbo et al., 2019 J Bone Joint Surg Am | Systematic review & meta-analysis | 35 studies; 48,873 primary TKAs | ~4.0% (pooled) | NR | First major pooled prevalence estimate of acquired idiopathic stiffness; BMI ≥30 associated with higher AIS prevalence (5% vs 2%; p=0.027). |
| Owen et al., 2021 J Arthroplasty | Single-institution retrospective cohort | 9,771 patients (12,735 knees) | 3.6% | 2.6% | Diabetes independently associated with AIS HR 1.49, 95% CI 1.11–1.99. BMI ≥30 inversely associated in multivariable model. |
| Rullán et al., 2022 Knee | Large state database (retrospective) | 100,613 primary TKAs | NR | 2.8% (1 in 36) | Higher MUA odds: younger age, female sex, Black/Hispanic ethnicity. Lower MUA odds with robotic/computer-assisted TKA vs conventional. |
| Patel et al., 2025 J Arthroplasty | Systematic review & meta-analysis | 53 studies involving 2,931,517 patients | NR | 3.6% (pooled) | Significant risk factors for MUA: prior knee surgery, younger age, Black race, smoking, ASA ≤2. Sex, obesity, diabetes NS in pooled analysis. |
| DeFrance et al., 2022 Orthopedics | Retrospective matched cohort | MUA vs matched controls | NR | NR (MUA cohort) | MUA patients had higher T2DM (13.0% vs 9.6%, p=0.025) and prior DVT (6.8% vs 3.8%, p=0.005). Non-infectious revision 7.3% vs 4.9% (p=0.034). |
| LaHaise et al., 2021 J Knee Surg | Retrospective cohort | 1,546 primary TKAs | NR | NR | Discharge flexion <65°: OR 17.57 for subsequent MUA vs >90°. Discharge ROM trajectory is a strong predictor of MUA need. |

AIS = acquired idiopathic stiffness; MUA = manipulation under anaesthesia; NR = not reported; NS = not significant; HR = hazard ratio; OR = odds ratio; DVT = deep vein thrombosis.

**Supplementary Table 2: MUA Outcomes: ROM Gains, Patient-Reported Outcomes, and Survivorship**

| Study (Author, Year, Journal) | Sample | Pre-MUA ROM | Post-MUA ROM | Mean ROM Gain | PROMs | Key Findings |
| --- | --- | --- | --- | --- | --- | --- |
| Akhtar et al., 2024 Meta-analysis J Arthroplasty | 14 studies 13,445 knees | 71.3° (early) 77.9° (delayed) | 103.0° (early) 96.1° (delayed) | 32.0° early 19.2° delayed (p<0.001) | Not separately reported | Early MUA (≤3 months): significantly greater ROM gain and lower revision/complication rates than delayed MUA. Complication rate: 4.9% (early) vs 10.3% (delayed). |
| Abdel et al., 2024 RCT (multicentre) J Arthroplasty | MUA arm (4–12 wks) | NR | NR | +46° immediate +28° at 6 wks +37° at 1 year | KSS, KOOS, PROMIS, SF-12 all improved | Adjunctive IV dexamethasone + celecoxib did NOT improve ROM or PROMs over standard MUA care. Highest-level RCT evidence for early MUA. |
| Debbi et al., 2025 Retrospective cohort HSS J | 543 MUAs | 78.0° | 112.8° | +34.8° | VAS, KOOS Jr, KSS, VR-12 physical significantly improved at ~15 months. VR-12 mental unchanged. | Pre-MUA flexion contracture ≥5° predicted repeat MUA and revision. 2-yr survivorship: 90.9% free from 2nd MUA; 92.4% free from revision for stiffness. |
| Roullet et al., 2026 Multicentre cohort Orthop Traumatol Surg Res | 344 patients 5-yr follow-up | 66.6° | 102.2° | +36.0° (p<0.001) | Satisfaction 77.5% at 5 years | Global complication rate 2.3%. Sub-group with 2nd MUA gained only +16.8° at 5 years (diminishing returns). Longest follow-up series. |
| Ruberto et al., 2026 Retrospective cohort J Arthroplasty | 103 patients (CSI vs no-CSI) | NR | NR | +42° immediate +16–17° at 4–6 wks (maintained) | No significant difference between CSI and non-CSI groups | Immediate ROM gain partially lost early then stabilises. No PJIs in either group. IACI did not improve outcomes vs MUA alone. |
| Sirignano et al., 2025 Retrospective matched J Knee Surg | MUA vs uncomplicated TKA controls | Flexion failure <105° at 6 wks (trigger) | NR | Not separately reported | KSS, WOMAC, KOOS JR, Forgotten Joint Score, satisfaction: NS vs controls at ≥2 yrs | MUA patients can achieve equivalent patient-reported satisfaction to uncomplicated TKA. Earlier threshold (105° at 6 wks) may be reasonable in young/active patients. |
| Haffar et al., 2022 Updated review of the literature Int Orthop | MUA, arthroscopic LOA, revision TKA cohorts | 78.7° (MUA group) | 99.7° (MUA group) | +21° (MUA) +40° (arthroscopic LOA) +28° (revision TKA) | Inconsistently reported across studies | MUA has best risk-benefit profile as first-line procedure. Arthroscopic LOA: 25% needed further treatment. Revision: greater burden and further interventions. |
| DeFrance et al., 2022 Retrospective matched Orthopedics | MUA vs matched TKA controls | NR | NR | NR | NR | Non-infectious revision rate: 7.3% (MUA) vs 4.9% (controls), p=0.034. 64% of post-MUA revisions were for persistent stiffness. MUA = independent risk marker for future revision. |

Studies listed in order of evidence level (RCT > meta-analysis > multicentre cohort > single-centre cohort > systematic review). ROM values represent mean degrees unless otherwise stated. Abbreviations: RCT = randomised controlled trial; ROM = range of motion; PROMs = patient-reported outcome measures; KSS = Knee Society Score; KOOS = Knee Injury and Osteoarthritis Outcome Score; WOMAC = Western Ontario and McMaster Universities Osteoarthritis Index; VAS = visual analogue scale; PROMIS = Patient-Reported Outcomes Measurement Information System; IACI = intra-articular corticosteroid injection; LOA = lysis of adhesions; SSI = surgical site infection; NR = not reported; NS = not significant; CSI = corticosteroid injection
